# The distribution of income is worse than you think: Including pollution impacts into measures of income inequality

**DOI:** 10.1371/journal.pone.0192461

**Published:** 2018-03-21

**Authors:** Nicholas Z. Muller, Peter Hans Matthews, Virginia Wiltshire-Gordon

**Affiliations:** 1 Department of Engineering and Public Policy, Tepper School of Business, Carnegie Mellon University, Pittsburgh, PA, United States of America; 2 National Bureau of Economic Research., Cambridge, Massachusetts, United States of America; 3 Department of Economics, Middlebury College, Middlebury, Vermont, United States of America; 4 Department of Economics, Hanken School of Economics, Helsinki, Finland; 5 ECONW, Portland, Oregon, United States of America; Universitat Jaume I, SPAIN

## Abstract

This paper calculates the distribution of an adjusted measure of income that deducts damages due to exposure to air pollution from reported market income in the United States from 2011 to 2014. The Gini coefficient for this measure of adjusted income is 0.682 in 2011, as compared to 0.482 for market income. By 2014, we estimate that the Gini for adjusted income fell to 0.646, while the market income Gini did not appreciably change. The inclusion of air pollution damage acts like a regressive tax: with air pollution, the bottom 20% of households lose roughly 10% of the share of income, while the top 20% of households gain 10%. We find that, unlike the case for market income, New England is not the most unequal division with respect to adjusted income. Further, the difference between adjusted income for white and Hispanics is smaller than expected. However, the gap in augmented income between whites and African-Americans is widening.

## Introduction

“It has been argued, for example, that if we measured income more comprehensively than we do, or if we measured it over periods longer than a year, a clearer trend toward equality would emerge.”

- Alan S. Blinder, 1980, [1].

With some exceptions, recent research on the distribution of income in the United States has supported two broad conclusions. The first is that with access to richer, more complete, data sets, we now have an accurate sense of that distribution, not least the income that accrues to the “one percent.” The second is that for most measures of dispersion, that distribution has become more unequal over time [2].

We challenge both of these conclusions, and advance two provocative claims; namely, that distribution is *much* more unequal than recent studies have suggested, but that it might also be becoming *more* equal over time. These surprising results are a consequence of our view that the improved measurement of income depends not just on better records of market income, but on more comprehensive definitions of income. In particular, our calculations are predicated on the decades old suggestion in Nordhaus and Tobin [3] that the official focus on output (and therefore income) of goods and services produced for, and sold in, organized markets is too restrictive.

We focus on one particular nonmarket outcome, the household costs associated with exposure to air pollution. In particular, we focus on mortality damages from fine particulate matter (PM_2.5_) and ground-level ozone (O_3_), because monitoring networks for these species are relatively broad and mortality risk accounts for the vast majority of adverse impacts from exposure to air pollution when expressed in monetary terms [4,5,6,7]. We compute individual damages by first calculating the fraction of mortality risk attributable to PM_2.5_ and O_3_ exposure, and then monetizing these risks, using techniques from a long and well-developed literature on the monetization of pollution damage [8,9,10,11,12]. Our first hypothesis asks whether inclusion of such impacts appreciably affects the distribution of resources across households in 2011. We then construct adjusted annual household income measures for 2011–2014. We focus on this time period because during these four years emission levels changed appreciably. As such, we are able to test our second central hypothesis: whether the distribution of our proposed measure of adjusted income is changing through time and how.

It is important to emphasize that our adjusted income measure deducts monetary costs attributable to exposure from reported household income. This specification of adjusted income is based on principles guiding the incorporation of environmental externality into measures of national income found in a longstanding literature consisting of both theoretical proposals and some examples of execution [6,13,14,15, 16,17]. Despite these earlier works, we are the first to link individual and household damage to market income and then to construct the distribution of this measure of adjusted income. This approach allows for direct comparison of measures of inequality based on market and adjusted income. It also enables us to work with a high degree of spatial resolution, to decompose results by regional and demographic groups, and to challenge a number of other stylized facts.

The incorporation of an environmental dimension into the analysis of inequality is itself not new [18]. Three particular strands of the literature are especially relevant here. The first concerns the distribution of emissions and exposure [19,20,21,22,23,24,25,26,27,28]. The second set of papers considers inequality as a determinant of emission rates or pollution outcomes [29,30,31]. A third literature gauges the impacts of environmental policies [32,33,34].

Our most important antecedents, however, are Boyce, Zwickl, and Ash [27] who estimate the Gini for exposure (not health impacts or monetary damages) and ratios of exposure among different ethnic and income groups, and Ash et al. [26], who conduct a related analysis examining exposure metrics among different population subgroups in U.S. urban areas. Neither augment market income with pollution damage nor do these prior researchers report measures of inequality in such incomes.

A few general observations about our methodological framework will provide some perspective on our results and, we hope, address some possible concerns. First, we view this research as another contribution to a well-established research program that, to echo Abraham ([17], p. 167), “widen[s] the coverage of national accounts to include all activities that generate … welfare, including those that take place beyond the market.” This is not to suggest that the extension of the national accounts is equivalent to the estimation of individual or collective utilities, but rather that a more complete reckoning of aggregate output and, under the rules of double entry bookkeeping, income is, at least in principle, a desirable outcome.

Second, while considerable energies in both the academic and public sectors have been devoted to the construction of “satellite accounts,” we believe that our particular focus on fine particulate matter and ground-level ozone is one of several natural “bridges” for the integration, however tentative, of market and non-market accounts. The use of environmental assets and services is, with household production and investments in health and human capital, a first order priority for those committed to the development of augmented accounts [16, 17] and, within this class, the effects of PM_2.5_ and O_3_ represent a substantial and practical choice. It is no more than a first step, however: it would be interesting to ask, for example, how the distribution of “full income” looks when, say, the costs of ground water pollution are included.

Third, our particular focus on the *distribution*, as opposed to level, of enhanced or augmented income finds echoes in Nordhaus’ [17] concept of “externality disaggregation,” in particular, the case in which the relevant transactions cross the boundary between market and non-market sectors. Drawing on one of his examples, to the extent that the market-measured profits of some industrial firms are higher than they otherwise would be because of an implicit transfer from the “non-market accounts” of households, the principles of national income accounting require that this be documented, not least because of the substantial implications for the distribution of income. Viewed from this perspective, our conclusion that relative to market income, the distribution of augmented or enhanced income embodies what amounts to a large transfer from the bottom quintile to the top acquires special meaning: the poor have provided a disproportionate share of non-market income subsidies.

Last, to forestall possible criticism, we observe, at the outset, that while rich households can and do expend some of their income on more expensive houses and other products associated with cleaner air, our estimates are *not* a consequence of double counting. We calculate residual or extant damage, given *reported pollution levels*, for all households, including affluent ones. Furthermore, because cross-sectional price variation may reflect spatial differences in public good provision, we use cost-of-living adjusted (COLA) incomes, but note that the COLA adjustment is even higher in high-damage communities.

## Results

We first use the results for 2011 to describe some cross-sectional patterns that are broadly consistent across each year in our sample period. Next, we consider how outcomes have evolved over time. In order to bolster our use of the top-coded market income data, we estimate a Gini coefficient for household income using all 1.2 million households in the IPUMS database for 2011. The estimated Gini, reported in Table A in S1 File (in the supplementary information) is, 0.476 in 2011, which is almost identical to the value of 0.475 reported by the U.S. Census [35] using uncensored data.

Table A in S1 File also reports the estimated Gini coefficients for the much smaller sample of households in counties with air pollution monitors and, for this subgroup, the estimated Gini for market income is 0.482. The distribution of income is not radically different for households in counties with pollution monitoring stations. However, the level of income is roughly 10% higher in monitored counties. Regulatory agencies often locate pollution monitoring stations in or nearby cities, so this difference is expected.

Table 1 reports the estimated Gini coefficients for market income, pollution damage, and adjusted income. While that for market income in monitored counties is 0.482, the Gini coefficient for pollution externality is 0.670, suggesting that damages are concentrated among a subset of households–more concentrated, in fact, than market income. If the households bearing a greater pollution burden were affluent households, the Gini coefficient for adjusted household income might be smaller than that for market income. As reported in Table 1, this Gini is 0.682, which suggests that when the definition of income includes air pollution, its distribution is much more unequal. We note, too, that while there were 191 households in the sample who reported negative market income in 2011, we calculate that about 22,000 households, about 5% of the sample, earned negative adjusted income. In sum, the difference in Ginis for market and adjusted income, (0.682 versus 0.482) suggests that pollution damage has a shockingly large and regressive effect on the distribution of economic resources. These Gini coefficients indicate that low-income households experience large monetary damages.

**Table 1:** Gini coefficients.

**All Households**							
**Year**	**Obs.**	**Market** **Income**	**Externality**	**Market Income** **- Externality**	**Share** **PM_2.5_**	**Share** **O_3_**	**Ratio** **(Adjusted/Market)**
**2011**	455,988	0.482 (0.001)^A^	0.670 (0.000)	0.682 (0.001)	0.793	0.207	1.416
**2012**	523,604	0.480 (0.001)	0.666 (0.001)	0.662 (0.001)	0.776	0.224	1.378
**2013**	540,137	0.484 (0.001)	0.664 (0.001)	0.652 (0.001)	0.781	0.219	1.348
**2014**	569,894	0.482 (0.001)	0.659 (0.001)	0.646 (0.001)	0.780	0.220	1.340
**White Households**							
**Year**	**Obs.**	**Market** **Income**	**Externality**	**Market Income** **- Externality**	**Share** **PM_2.5_**	**Share** **O_3_**	**Ratio** **(Adjusted/Market)**
**2011**	343,824	0.472 (0.001)	0.675 (0.001)	0.671 (0.001)	0.791	0.209	1.422
**2012**	400,412	0.471 (0.001)	0.670 (0.001)	0.652 (0.001)	0.774	0.226	1.386
**2013**	413,625	0.475 (0.001)	0.667 (0.001)	0.642 (0.001)	0.779	0.221	1.352
**2014**	434,219	0.473 (0.001)	0.663 (0.001)	0.635 (0.001)	0.779	0.221	1.345
**African American Households**							
**Year**	**Obs.**	**Market** **Income**	**Externality**	**Market Income** **- Externality**	**Share** **PM_2.5_**	**Share** **O_3_**	**Ratio** **(Adjusted/Market)**
**2011**	60,630	0.484 (0.001)	0.670 (0.001)	0.750 (0.004)	0.803	0.197	1.549
**2012**	67,880	0.484 (0.001)	0.670 (0.001)	0.710 (0.003)	0.787	0.213	1.469
**2013**	69,285	0.484 (0.001)	0.668 (0.001)	0.687 (0.003)	0.789	0.211	1.420
**2014**	75,199	0.484 (0.001)	0.662 (0.002)	0.693 (0.003)	0.793	0.207	1.433

A = bootstrap standard error in parenthesis.

This begs the question, why are damages so regressive? The calculation of damages–see Eqs (1) and (2) in the methods section, below–depends on ambient pollution levels, baseline mortality rates and the monetary value attributed to mortality risk (the Value of a Statistical Life, or the VSL). Because our baseline specification holds the VSL fixed across households, the distribution of damages must reflect variation in exposure and mortality rates. To explore these possible sources of inequality, we compute the Gini coefficients for ambient concentrations of PM_2.5_ and O_3_. The fitted coefficients are 0.113 (se = 0.001) and 0.091 (se = 0.001) for PM_2.5_ and O_3_, respectively. The estimated Gini coefficient for baseline mortality risk is 0.712 (se = 0.002). Our surprising conclusion, therefore, is that while the distribution of individual exposure is reasonably equal, the distribution of risk is not. The latter drives the result for adjusted income. We note, in the discussion section below, that other pollutants are distributed differently across space and among communities. Our focus on PM_2.5_ and O_3_ stems from the repeated finding that they contribute the greatest share of damages among all pollutants regulated by the Clean Air Act [4,5].

To provide another perspective on these results, S1 and S2 Figs in the supplementary information (S1 File) present scatter plots for pollution levels and mortality risks against household income using the 2011 sample. Wealthier households are often exposed to lower levels of air pollution. This claim is also supported by Table F in S1 File (the supplementary materials), which shows differences in annual average PM_2.5_ by income quantile. Furthermore, the exposure is less harmful among higher income groups because mortality rates (baseline risk) are also lower for richer households (see S2 Fig). Or, in more provocative terms, the households least able to bear the costs of air pollution are exposed to more of it. The same logic also suggests that damages increase with the age of the exposed, a result previously reported in the literature [9]. S3 Fig confirms this result in the present context.

Just how large is the increase in Gini coefficients from 0.482 to 0.682? Subject to caveats about the comparison of Ginis with and without negative incomes, one obvious reference point is international differences. According to World Bank estimates, our pollution-adjusted Gini coefficient is greater than the Gini coefficient on market income for Haiti and South Africa [36]. Another natural comparison is the difference between pre-tax and post-tax and transfer distributions in the United States. Using data for the same year (2011), the OECD [37] reports that the pre-tax Gini was 0.508 (which is a little higher than our own estimate and that published by the U.S. Census) while the after-tax Gini was 0.389. This is a 31% difference, which is 10 percentage points less than the difference in market and adjusted income Ginis that we calculate. Differences in exposure to local air pollution effectively erase the gains in equality made through progressive income taxation, social security and other government programs. This result suggests that policymakers should also view environmental policies as redistributive tools.

Other measures of income distribution exist, of course, and Table 2 compares the estimated shares for market and adjusted household incomes. If we limit the sample to observations that can be matched to pollution monitoring stations, in 2011, the bottom quintile earns about 3% of market income, while the top quintile earns just over 50%, a ratio of 16.7 to 1, consistent with other studies and further confirmation that the distribution of income is indeed lopsided.

**Table 2:** Income shares.

	**2011**		**2012**	
**Income Quantiles**	**Market Income**	**Adjusted Income**	**Market Income**	**Adjusted Income**
**< 20**	0.031 (0.0000891)^A^	-0.074 (0.0005778)	0.031 (0.0000843)	-0.063 (0.0004813)
**20–40**	0.082 (0.0001497)	0.061 (0.0001949)	0.083 (0.0001393)	0.063 (0.0001784)
**40–60**	0.142 (0.0001941)	0.143 (0.0002525)	0.143 (0.0001831)	0.143 (0.0002298)
**60–80**	0.229 (0.0002467)	0.255 (0.0003619)	0.229 (0.0002296)	0.252 (0.0003267)
**80–100**	0.516 (0.0005313)	0.614 (0.0007293)	0.514 (0.0004989)	0.605 (0.0006556)
	**2013**		**2014**	
**Income Quantiles**	**Market Income**	**Adjusted Income**	**Market Income**	**Adjusted Income**
**< 20**	0.031 (0.0000853)	-0.055 (0.000439)	0.031 (0.0000817)	-0.053 (0.000411)
**20–40**	0.083 (0.0001373)	0.064 (0.0001727)	0.083 (0.000135)	0.064 (0.0001684)
**40–60**	0.142 (0.0001804)	0.141 (0.0002241)	0.142 (0.0001772)	0.142 (0.000218)
**60–80**	0.226 (0.0002306)	0.247 (0.0003174)	0.227 (0.0002233)	0.248 (0.0003066)
**80–100**	0.518 (0.0005012)	0.603 (0.0006348)	0.517 (0.0004879)	0.599 (0.0006131)

A = standard error in parenthesis.

Table 2 reports the same shares for adjusted incomes in 2011. The lowest quintile now earns a *negative* share (-7%) of national income; for a substantial number of households in this group, adjusted income is negative. The share of the top quintile increases, however, from 52% to 61%. In short, the bottom quintile loses 10 percentage points while the top quintile gains about 10 percentage points. Pollution damage therefore results in a symmetric and bracingly regressive reallocation of income from the bottom quintile to the top. Consistent with an observation made in the introduction, one way to view this outcome is as a transfer of non-market income. The incorporation of damages from pollution does not change the shares of the middle three quintiles much, however. This implies that the mortality impacts from exposures to PM_2.5_ and O_3_ are equitably distributed across households in these income percentiles.

Our income share analysis therefore supports the conclusion that the adjustment for pollution damages functions like a regressive tax. It also reveals that the redistribution is itself concentrated. That is, it is not as though the increased share that accrues to the top quantile comes in equal measure from the bottom four quantiles: rather, the burden of this regressive pseudo-tax falls squarely on the shoulders of the poorest 20% of households.

### Changes from 2011–2014 and their decomposition

In addition to 2011, Table 1 reports, among other things, the overall Ginis for market income, damages and adjusted income between 2012 and 2014. Consistent with U.S. Census calculations, there has been little or no change in the market income Gini during this period (see Table A in S1 File). There has been a substantial decrease in the Gini for adjusted income, however, from 0.682 (se = 0.001) to 0.646 (se = 0.001). In other words, and in contrast to some public narratives revolving around market income, the distribution of adjusted income has become *more* egalitarian since 2011. One reason for this is found in the Gini for damages: the costs of exposure to air pollution became more equally distributed, as reflected in the modest decline of the damage Gini from 0.670 (se = 0.0005) to 0.659 (se = 0.0005). The share decomposition reveals that, while the dominant share of inequality in pollution damage stems from exposure to PM_2.5_, this share is falling relative to O_3_ between 2011 and 2014. For 2011 through 2014, O_3_ levels averaged about 28 parts per billion. In contrast, PM_2.5_ concentrations fell by 10% from 10.2 to 9.3 (ug/m^3^). These significant reductions in PM_2.5_ concentrations imply reductions in emissions of primary PM_2.5_ in addition to precursors such as sulfur dioxide, nitrogen oxides, ammonia, and non-methane hydrocarbons. Another potentially important factor driving the reduction in the adjusted income Gini coefficients was the enactment of the Affordable Care Act (ACA). This large policy intervention likely affected baseline health status (mortality rates) primarily among the previously uninsured. Communities for which the individual mandate was a binding policy change consist of low-income populations without access to insurance and relatively healthy persons that elected not to purchase insurance policies. To the extent that the ACA affected the adjusted income Gini (an empirical test we leave for future work), it must have predominantly improved the health status among the former—low-income populations without access to insurance.

Table 2 displays the income shares for 2012 through 2014. The result that pollution damage transfers economic resources from the bottom 20% of household units to the top 20% is remarkably robust. The percentile shares of market income are quite stable across these four years with the bottom 20% earning about 3% and the top 20% earning 51% of income. In terms of adjusted income, recall from the discussion above that, in 2011, the bottom 20% earned -7%. Importantly, this percentage creeps closer to zero–implying fewer negative income households. Concomitantly, the share of adjusted income accruing to the top 20% fell from 61.4% in 2011 to 59.9% in 2014. Small changes indeed, however, these support the attenuation of the adjusted income Gini coefficients over time reported in Table 1. Adjusted income becomes equal between 2011 and 2014.

### Spatial decomposition

**Fig 1:**
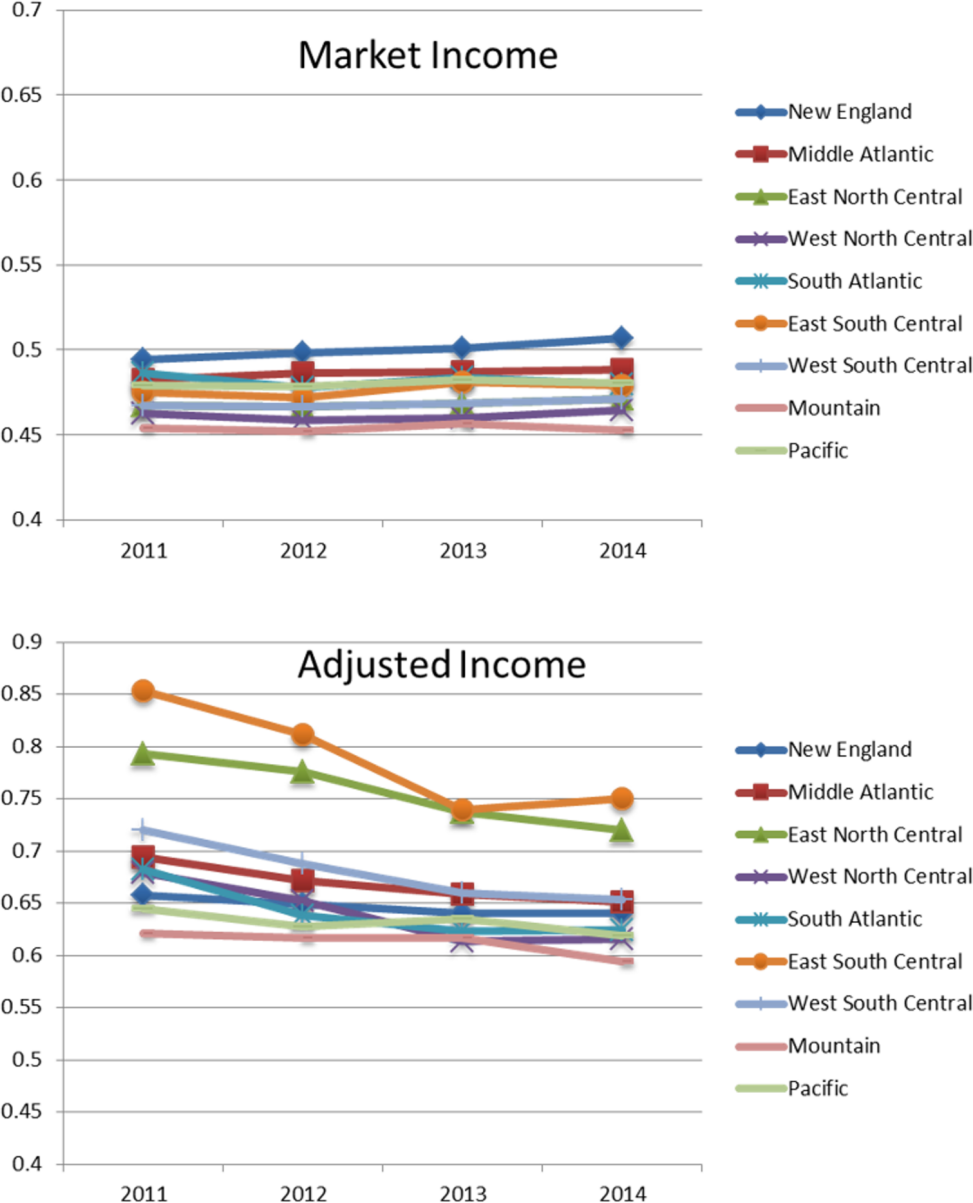
Market and adjusted income Gini coefficients by Census region.

Fig 1 presents the Gini coefficients for market income (top panel) and adjusted income (bottom panel) across census divisions. Consistent with official Census data, there are modest differences in market income inequality. The Gini is highest in New England (0.494 in 2011, increasing to 0.507 in 2014) and the Middle Atlantic (0.482 in 2011 and marginally higher, 0.489, by 2014), and lowest in the Mountain division (0.454 in 2011, and almost identical, 0.453, in 2014).

The divisional Gini coefficients for adjusted income challenge received wisdom. Viewed from this perspective, the two most unequal regions are, by substantial margins, East South Central (Alabama, Kentucky, Mississippi and Tennessee) and East North Central (Illinois, Indiana, Michigan, Ohio and Wisconsin), where the Ginis in 2011 were 0.854 and 0.793, respectively. On the other hand, these were also the divisions in which the Gini decreased most, to 0.750 and 0.720, respectively. In contrast, in terms of adjusted incomes, New England (0.657 in 2011 and 0.640 in 2014) is one of the most egalitarian divisions, but has not become substantially more equitable over this period. In short, reckoned in terms of adjusted income, the “heartland,” and not the urban northeast, is the most unequal region of the country, but is becoming somewhat more egalitarian. While a thorough exploration of the causes of regional disparities in the distribution of adjusted income is beyond the scope of this paper, we note that the East South Central and East North Central divisions contain considerable coal-fired power generation capacity and, especially for the East North Central division, heavy manufacturing. Both of these types of production produce copious amounts of air pollution.

### Racial and ethnic decomposition

We provide two perspectives on the racial and ethnic dimensions of income distribution, both of which also challenge the conventional wisdom focused on the market income distribution. We first consider between group differences at a national level. S4 Fig depicts the ratios of white to African-American and white to Hispanic median household incomes for both market and adjusted income. There is a 32% difference in the median market incomes of white and Hispanic households in 2011 and this spread decreases, a little, to 29% in 2014. This is one of the few cases we observe in which the difference in market income exceeds that of adjusted income; adjusted income is 29% higher in 2011 and the difference shrinks to 21%, in 2014.

The differences between whites and African-Americans are even more concerning, however. As is well known, there is an almost 65% difference in household incomes in 2011 and this decreased, to about 62%, by 2014. The difference in adjusted incomes is both larger and increasing: the median white household earned 67% more in 2011, including the costs of air pollution exposure, and this increased to 73% by 2014. In short, standard income measures *understate* the differences between whites and African-Americans, and the extent to which the problem is becoming worse.

The second perspective on racial income inequality is on the differences between market and adjusted Ginis within groups. The bottom of Table 1 reports these measures for white and African-American households. Table D in S1 File (in the supplementary information) covers Asian and Hispanic households. We first note that the distribution of market income within each group remained more or less unchanged. The distribution was a little more unequal for African-Americans (0.484 in 2011 and 0.484 in 2014) than whites (0.472 in 2011 and 0.473 in 2014), which, in turn, was a little more unequal than that for Hispanics (0.445 in 2011 and 0.441 in 2014).

The distribution of adjusted income within these populations is another matter entirely. The Gini for African-Americans was much higher in 2011 (0.750, se = 0.004) than for either whites (0.671, se = 0.001) or Hispanics (0.577, se = 0.003) *and* experienced a substantial decrease, to 0.693 (se = 0.003) in 2014. The adjusted income Gini for whites also decreased, although not quite as much, to 0.635 (se = 0.001), while that for Hispanics experienced a still smaller decline, from 0.577 to 0.551 (se = 0.002). Once more, there is something to be learned from the distribution of air pollution: while damage Ginis for the three groups are quite similar, those for African-Americans and whites, declined, while that for Hispanics was more or less unchanged. The improvement in the overall distribution of income reflects, in considerable measure, improvements for whites and African-Americans, among whom the distribution of damages has become more equal. Furthermore, all of this can be attributed to the more equitable distribution of PM_2.5_-related costs.

### Sensitivity analysis

Table E in S1 File (the supplementary materials) displays the results of a sensitivity analysis for 2011. We note first that the Gini coefficient for damages varies from 0.672 (se = 0.001) to 0.669 (se = 0.001) and so is robust with respect to parameter choice. The estimated Gini coefficient for adjusted income is more sensitive, however, to these parameters: while the Gini in the default case is 0.682 (se = 0.001), it increases to 1.032 (se = 0.003) when the concentration-response function for mortality effects from PM_2.5_ exposure reported by LePeule et al., [38] is used. (Note that because our measure allows for negative values, the usual interpretation of a Gini of unity does not hold.) If anything, however, this finding only reinforces one of our basic conclusions, namely, that the distribution of adjusted income is much more unequal than that of market income. If instead of the $10.2 million VSL one uses the $3.3 million VSL some in the literature [39] have recommended, the Gini falls to 0.536 (se = 0.001), which tempers but does not change this conclusion. Likewise, if the VSL is adjusted by income of the exposed population, the Gini becomes 0.618 (se = 0.001), still much higher than the market income benchmark of 0.482. (The appendix discusses the technique used to make this adjustment of the VSL.) Suffice it to note here that the income-VSL elasticity is positive. The implication of this is that households toward the top of the market income distribution are attributed a willingness-to-pay (WTP) for mortality risk reduction that is higher than for other households. Importantly, this is not a normative judgement regarding the value of lives. Rather it is a reflection of the fact that WTP for risk reductions is constrained by income.

The bottom panel in Table E in S1 File shows the variation in the calculated income shares under alternative damage computation scenarios. The pattern of a transfer from the bottom quintile to the top proves robust: for example, when the $3.3 million VSL is used, the income share of the bottom quintile falls from 3.1% to 0.6%, while the share of the top quintile rises from 51% to 55%. Using the higher concentration-response function produces a more dramatic reallocation of income, but, once more, our basic conclusion is unaffected. The gains at the top of the income distribution come at the expense of those at the very bottom. When the VSL is adjusted by income, the redistribution is less dramatic but the symmetry of gains and losses in the top and bottom quintiles is preserved. In summary, the sensitivity analysis affects the degree to which the distribution of adjusted income differs from that for market income. Alternative values for key parameters do not affect the manner in which the distributions differ.

## Discussion

This analysis challenges common perceptions of the distribution of income by estimating an augmented measure of income inclusive of pollution damage. This tack builds on a much earlier literature [3,13] to make, essentially, two new points: (1) adjusted income of this sort is more unequal than reported market income, and (2) adjusted income is becoming *less* unequal over time. Within these broadly important results, we also report several more nuanced, yet perhaps equally compelling findings. First, the spatial pattern of inequality in adjusted income differs from that of market income; states in the colloquial heartland exhibit very high degrees of adjusted income inequality whereas states in the urban northeast tend to show the highest extent of market income inequality. However, in the heartland, inequality in adjusted income fell more rapidly than market income. Second, African-American communities exhibit radically higher inequality in adjusted income relative to market income and relative to other racial groups. Among white and African-American households, the measure of inequality in adjusted income fell more rapidly than in households comprised of different races. Third, pollution damage acts like a highly regressive and remarkably concentrated tax. The inclusion of pollution damages reallocates income from the bottom 20% of households to the top 20%. The middle quintiles are essentially unaffected. This result remains robust over time and over different ways of measuring damages.

We note several caveats to our findings. First, as with any attempt to measure pollution damages, our estimates embody considerable uncertainty. For example, both the connection between exposure and mortality [38,40] and mortality risk and monetary damages [41] are statistically estimated relationships. Hence, while we use reported mean parameter values, there is statistical variability around these measures of central tendency. Second, as noted above, we employ top-coded market income data. We leave issues associated with estimation of adjusted income for the extreme right-hand tail of the household income distribution to future work. Third, the extant monitoring networks for both O_3_ and PM_2.5_ are spatially far from complete. Thus, our results are beholden to the design of the pollution monitoring networks and the truncated sample of households in counties with such devices. Finally, we recognize that the focus here is on one set of environmental pollutants. There are, of course, many others including airborne toxics, water pollutants, toxins in the soil, and adverse effects from climate change. Our focus lies on O_3_ and PM_2.5_ because of the large damages associated with these species reported in the literature [4,5,7] and because of relatively rich monitoring data. However, the inclusion of adverse effects due to other pollutants may substantively affect our results.

We conclude by arguing that our results reported herein are intended to shift the debate about income inequality from a singular focus on market income to one that is inclusive of phenomena that the economics literature has recognized are important to national welfare for over 40 years.

## Methods

The calculation of Gini coefficients for market income is based on the 2011–14 American Community Surveys (ACS), as provided by the Minnesota Population Center’s Integrated Public Use Microdata Series (IPUMS) [42]. We use total household income, which is the sum of wage/salary income, total personal income, and dividend income. The data are top-coded for identity protection. Specifically, any observation above the 99.5^th^ percentile in each state is replaced with the mean value of all incomes above the 99.5^th^. We do not correct for topcoding. To bolster this approach we compute Gini coefficients (for market income) using the top-coded data and then compare these to the Gini coefficients reported by the U.S. Bureau of the Census for each of the above years in the sample. Table A in S1 File (the supplementary information) displays the results of this exercise. For each data year from 2011 to 2014, there are just over 1 million households with total household income and county location identifiers, which are used to match households to air pollution readings.

All-cause mortality rate data for the same data years are from the U.S. Centers for Disease Control and Prevention [43]. These data are specific to 15 age groups (determined by the CDC) and spatially resolved at the county level. The data are plagued by numerous missing age-county observations, however, and the CDC marks as unreliable any estimate of the mortality rate based on fewer than 20 documented deaths. We retain only reliable observations as determined by CDC.

The United States Environmental Protection Agency’s (USEPA) Air Quality System (AQS) monitoring network [44] provides the air pollution data. The monitoring stations collect hourly readings of environmental pollutants such as tropospheric ozone (O_3_), sulfur dioxide, nitrous oxide, and particulate matter. For this study, we use data on O_3_ and PM_2.5_. For a variety of reasons, the monitors do not provide complete spatial coverage. Monitors tend to be located in areas either suspected of being out of compliance or with a track record of being out of compliance with ambient standards. In 2011, there were about 1,300 O_3_ monitors and 900 PM_2.5_ monitors. For PM_2.5_, the monitor count increased to 923 in 2013 and 975 in 2014. For O_3_, the active monitor count climbed to 1,314 in 2013 before falling back to 1,283 in 2014. Because households are coded by county, and mortality rates are reported by county, we average the air pollution data to the county level and then match, by county, to households and mortality rates.

### Empirical calculations

Among the damages associated with exposure to local air pollution, increased mortality risk contributes the largest share of monetary damage [4,5,6,9]. As such, the present analysis calculates the attributable share of extant mortality risk that is due to exposure to both PM_2.5_ and O_3_. To do so we employ the following approach, which is standard in both the academic literature and it is employed by the USEPA in its Regulatory Impact Analyses for air pollution and in the legislatively mandated benefit-cost analyses of the entire Clean Air Act [4,5,6,12]. Let $M_{i,t}^{p}$ denote the mortality risk for individual (i), at time (t), due to exposure to pollutant (p).

$$M_{i,t}^{p}=\gamma_{a,r,t}\left(1-(\frac{1}{exp(\beta^{p}C_{r,t}^{p})})\right)$$ (1)

where: γ_a,r,t_ = baseline all-cause mortality rate, age-cohort (a), location (r), time (t)

*β*^p^ = empirically estimated concentration-response parameter for pollutant (p).

*C*^p^_r,t_ = ambient concentration of pollutant (p), location (r), time (t).

Because of the use of micro data (the IPUMS), we calculate individual mortality risk, rather than spatially aggregated risk among populations within a grid-cell or county as is more commonplace in the literature. We note that, as expression (1) implies, we attribute population age-cohort mortality risk to individuals within that cohort, by county. Also, note that the income attributed to individuals in the sample is total household income.

Another implication of expression (1) warrants particular attention: two individuals exposed to the same levels of air pollution will be assigned different mortality risks if they are members of groups with different baseline mortality rates. In somewhat different terms, spatial variation in risk reflects both variation in exposure to air pollution and, to a surprising degree, variation in baseline mortalities, itself a reflection of variation in baseline demographic characteristics.

The estimated concentration-response coefficients are obtained from the epidemiological literature. Specifically, we use the reported coefficients that link exposure to PM_2.5_ to adult mortality risk from Krewski et al., [40] and LePeule et al., [38]. For the mortality effects of exposure to O_3_, we use the results from Bell et al., [45]. USEPA [4, 5] and academic researchers [7, 46, 47] often use these studies.

To monetize mortality risk, we adopt the Value of a Statistical Life (VSL) approach [41], which exploits individual trade-offs between income and risk, as inferred from market transactions (like the trade-off between wages and on-the-job mortality risk) or highly structured surveys. While there is wide variation in VSL estimates in the literature, we avoid the usual debates and adopt a measure that is commonly used by regulatory agencies and many academic researchers. In particular, the VSL we adopt is that used by the USEPA, based on from 26 studies that used a mixture of approaches: in a recent regulatory impact analysis of O_3_, it assumed a VSL of $8.3 million (for the 1990 income level) or $9.5 million (for the 2010 income level), both expressed in 2011 prices [48]. For the purposes of the present analysis, we adjust the $9.5 million VSL according to the nominal income levels in 2011–2014 using the EPA’s income-VSL elasticity of 0.4 [49]. This yields a VSL in 2011 of $10.2 million. In a sensitivity analysis, we use the $3.3 million VSL reported by Kochi et al., [39]. We conduct the sensitivity analysis only in the year 2011. Hence, the annual adjustments are not applied to the alternative VSL.

Given these considerations, monetary damage across pollutants, incurred by an individual (i) is given by:

$$D_{i,t}=\sum_{p=1}^{P}\left(VSL\times M_{i,t}^{p}\right)$$ (2)

As the notation hints, our default specification adopts the egalitarian approach of using the same VSL across populations of all ages, incomes, races and genders [4,5]. However, in our sensitivity analysis we allow the VSL to vary across income groups, on the grounds that it is a willingness-to-pay (WTP) measure that almost certainly varies with income. (We would remind readers, however, that even in its limited role as a robustness check, this is not a normative claim about differences in the “economic values” but rather the recognition that demand is income-constrained in the real world.) To perform this adjustment, we use the VSL-income elasticity of 0.4 reported in USEPA [49].

Adjusted household income for individual (i) at time (t) is denoted ($I_{i,t}^{a}$), and defined to be the difference between market income ($I_{i,t}^{m}$) and damage $\left(D_{i,t}):\;(I_{i,t}^{m})‑(D_{i,t}\right)$. It is important to note that while it is quite rare for households to have negative market income, negative adjusted income will be (relatively) common: the poorest households will often experience damages that exceed their incomes.

We use the algebraic equivalent of the generalized covariance-based Gini coefficient [50], as implemented in Stata’s “sgini” package [51]:

$$G(Y_{t})=-2Cov\left(\frac{Y_{t}}{\mu (Y_{t})},\left(1-F(Y_{t})\right)\right)$$ (3)

where *Y*_*t*_ is household income, market or adjusted, in period t, *μ*(*Y*_*t*_) is its mean and F(*Y*_*t*_) is its distribution function. The generalization admits negative incomes, which, while it changes the range of admissible values and complicates interpretation, is essential for the reason just outlined: adjusted income is sometimes less than zero. The standard errors for our estimated Gini coefficients are bootstrapped.

## Supporting information

S1 File Tables A-F.
(DOCX)

S1 Fig PM_2.5_ levels and household income. (TIF)

S2 Fig Mortality rate and household income. (TIF)

S3 Fig Damages by age of persons in household. (TIF)

S4 Fig Ratios of market and adjusted income across racial and ethnic groups. (TIF)
