# Supplementary material for: The distribution of income is worse than you think: Including pollution impacts into measures of income inequality
Source: PLoS One. 2018 Mar 21;13(3):e0192461. doi: 10.1371/journal.pone.0192461 (PMC5862398; doi:10.1371/journal.pone.0192461)
Supplement: S1 File — Tables A-F. (DOCX) [file pone.0192461.s001.docx]

**The Distribution of Income Is Worse Than You Think: Including Pollution Impacts Into Measures of Income Inequality.**

Nicholas Z. Muller

Peter H. Matthews

Virginia Wiltshire-Gordon

**Supplementary Information.**

Table A displays the summary statistics for the income data used in the empirical analysis. Inclusive of all observations, median income in 2011 was $49,400; this is nearly identical to the estimate provided by the U.S. Census Bureau of $50,054 (Census, 2012). When restricted to the counties with air pollution monitors, the median income among these 24,000 households is $54,000. Among all households, median income increased to just under $55,000 in 2014. Again, this is quite similar to income reported by the U.S. Census: $53,657. The right-skewed nature of the income distributions is also evident in that the means are much larger than the medians for all years. The estimated Ginis using topcoded IPUMS data and the “official” Ginis reported by the Census are also given in table A. For each year these two sets of Gini coefficients are quite similar.

Table B reports household income, ambient measures of O_3_ and PM_2.5_, damage from exposure to both pollutants, and damage expressed as a fractional share of income for 2011. Across all regions, O_3_ levels are about 29 ppb on average, while PM_2.5_ concentrations tend to be about 10 ug/m^3^. Median damage due to exposure to these levels of pollution amount to $2,928. This is equivalent to roughly 5% of household income.

Table B also reports regional summary statistics. Median income is highest in the east region, though the regional differences are within $3,000, or about 5% of national median income. Ambient pollution levels also vary regionally within a fairly narrow range. The lowest estimate of household damage from exposure is $2,435 in the western region. This comprises 4% of household income. The highest household damage is estimated to be $3,947 in the east region. Again, this amounts to 5% of income.

Table C decomposes the empirical summary statistics by racial category. There are wide disparities in median income across racial groups. In white households, median income is reported to be $59,200. Among Asian households, median income is over $70,000. And, in African American homes, median income is just $35,980. The reported O_3_ and PM_2.5_ levels do not systematically vary across racial groups. . Damages in African American households are estimated to be $2,967, whereas among Asian homes, damages are just $1,683. Further, damages, and income are negatively correlated. That is, damages comprise 8% of income for African Americans, 5% of white household income, and just 2% for Asians. These summary statistics are a leading indicator of the unequal distribution of pollution damage across American households.

**Sensitivity Analysis**

Table E displays the results of a sensitivity analysis for 2011. We note first that the Gini coefficient for damages varies from 0.672 (se = 0.001) to 0.669 (se = 0.001) and so is robust with respect to parameter choice. The estimated Gini coefficient for adjusted income is more sensitive, however, to these parameters: while the Gini in the default case is estimated to be 0.682 (se = 0.001), it increases to 1.032 (se = 0.003) when the concentration-response function for mortality effects from PM_2.5_ exposure reported by LePeule et al., [38] is used. (It should be noted, however, that because our measure allows for negative values, the usual interpretation of a Gini of unity does not hold.) If anything, however, this sensitivity only reinforces one of our basic conclusions, namely, that the distribution of adjusted income is much more unequal than that of market income. If instead of the $10.2 million VSL one uses the $3.3 million VSL some in the literature [39] have recommended, the Gini falls to 0.536 (se=0.001), which tempers but does not change this conclusion. Likewise, if the VSL is adjusted by income of the exposed population, the Gini becomes 0.618 (se=0.001), still much higher than the market income benchmark of 0.482.

The bottom panel in Table E shows the variation in the calculated income shares under alternative damage computation scenarios. The pattern of a transfer from the bottom quintile to the top proves robust: for example, when the $3.3 million VSL is used, the income share of the bottom quintile falls from 3.1% to 0.6%, while the share of the top quintile rises from 51% to 55%. Using the higher concentration-response function produces a more dramatic reallocation of income, but, once more, our basic conclusion is unaffected. The gains at the top of the income distribution come at the expense of those at the very bottom. When the VSL is adjusted by income, the redistribution is less dramatic but the symmetry of gains and losses in the top and bottom quintiles is preserved.

**Table A: Summary Statistics, Household Income.**

| All Households | | | | |
| --- | --- | --- | --- | --- |
|  | 2011 | 2012 | 2013 | 2014 |
| Mean | 68,616^A^ | 71,445 | 75,227 | 76,742 |
| Median | 49,400 | 51,000 | 53,300 | 54,700 |
| Max | 3,563,100 | 2,087,000 | 2,090,000 | 1,951,000 |
| Min | -18,490 | -17,300 | -19,770 | -14,700 |
| SD | 73,368 | 76,100 | 82,109 | 83,350 |
| Count | 1,204,830 | 1,207,725 | 1,211,264 | 1,218,240 |
| Gini Est. | 0.476 | 0.475 | 0.479 | 0.478 |
| Census Gini^B^ | 0.475 | 0.476 | 0.481 | 0.480 |
| Households in Counties with Pollution Monitors | | | | |
|  | 2011 | 2012 | 2013 | 2014 |
| Mean | 76,313 | 77,993 | 81,867 | 83,816 |
| Median | 54,000 | 55,055 | 57,700 | 59,800 |
| Max | 1,760,400 | 1,711,330 | 2,052,500 | 1,802,000 |
| Min | -14,800 | -15,000 | -14,000 | -14,000 |
| SD | 81,748 | 83,542 | 89,301 | 91,028 |
| Count | 455,988 | 523,604 | 540,137 | 569,894 |
| Gini Est. | 0.482 | 0.480 | 0.484 | 0.482 |

**Table B: Summary Statistics by Region for 2011.**

| Region | Household  Income ($) | O_3_  (ppb) | PM_2.5_  (ug/m^3^) | Per Capita  Damage($) | Damage/  Income |
| --- | --- | --- | --- | --- | --- |
| East | | | | | |
| Mean | 82,533.49 | 27.23 | 10.11 | 10,142.75 | 0.67 |
| Median | 58,800.00 | 27.33 | 9.80 | 3,046.04 | 0.05 |
| SD | 91,174.51 | 3.70 | 1.62 | 19,527.36 | 22.24 |
| Min. | -14,800.00 | 18.68 | 5.68 | 36.13 | -100.76 |
| Max. | 1,760,400.00 | 35.51 | 14.45 | 146,673.40 | 4,961.93 |
| Obs. | 101,199 | 101,199 | 101,199 | 101,199 | 100,055 |
| Midwest | | | | | |
| Mean | 66,427.60 | 29.60 | 10.74 | 11,504.15 | 0.85 |
| Median | 49,000.00 | 30.22 | 10.77 | 3,946.50 | 0.07 |
| SD | 67,394.99 | 2.50 | 1.72 | 21,197.38 | 25.17 |
| Min. | -12,500.00 | 22.32 | 6.07 | 48.23 | -35.28 |
| Max. | 1,077,600.00 | 36.32 | 14.33 | 149,680.80 | 4,324.89 |
| Obs. | 65,084 | 65,084 | 65,084 | 65,084 | 64,237 |
| South | | | | | |
| Mean | 73,040.27 | 29.21 | 10.65 | 9,727.09 | 0.76 |
| Median | 50,000.00 | 29.45 | 10.70 | 3,302.01 | 0.06 |
| SD | 79,911.57 | 2.76 | 1.21 | 18,151.12 | 40.37 |
| Min. | -14,800.00 | 22.97 | 7.94 | 41.03 | -803.54 |
| Max. | 1,284,000.00 | 36.19 | 13.57 | 146,866.10 | 8,405.55 |
| Obs. | 105,474 | 105,474 | 105,474 | 105,474 | 103,946 |
| West | | | | | |
| Mean | 78,115.33 | 28.47 | 9.86 | 8,055.39 | 0.92 |
| Median | 55,600.00 | 26.57 | 10.36 | 2,435.17 | 0.04 |
| SD | 81,625.99 | 5.51 | 2.23 | 16,339.89 | 68.43 |
| Min. | -12,400.00 | 18.05 | 3.87 | 32.12 | -374.87 |
| Max. | 1,397,000.00 | 45.12 | 17.06 | 160,565.40 | 23,813.74 |
| Obs. | 180,997 | 180,997 | 180,997 | 180,997 | 178,785 |
| All Regions | | | | | |
| Mean | 76,240.45 | 28.53 | 10.23 | 9,407.16 | 0.82 |
| Median | 54,000.00 | 28.42 | 10.38 | 2,927.58 | 0.05 |
| SD | 81,784.32 | 4.31 | 1.87 | 18,297.96 | 49.53 |
| Min. | -14,800.00 | 18.05 | 3.87 | 32.12 | -803.54 |
| Max. | 1,760,400.00 | 45.12 | 17.06 | 160,565.40 | 23,813.74 |
| Obs. | 452,754 | 452,754 | 452,754 | 452,754 | 447,023 |

All statistics from counties with both pollution monitors and income data.

**Table C: Summary Statistics by Racial Category for 2011.**

| Region | Household  Income ($) | O_3_  (ppb) | PM_2.5_  (ug/m^3^) | Per Capita  Damage($) | Damage/  Income |
| --- | --- | --- | --- | --- | --- |
| White | | | | | |
| Mean | 82,183.97 | 28.79 | 10.13 | 10,232.43 | 0.85 |
| Median | 59,200.00 | 28.99 | 10.27 | 3,225.14 | 0.05 |
| SD | 85,839.50 | 4.28 | 1.93 | 19,455.83 | 54.37 |
| Min. | -14,800.00 | 18.05 | 3.87 | 9.59 | -803.54 |
| Max. | 1,760,400.00 | 45.12 | 17.06 | 160,565.40 | 23,813.74 |
| Obs. | 343,824 | 343,824 | 343,824 | 343,824 | 340,533 |
| African American | | | | | |
| Mean | 50,143.19 | 28.13 | 10.67 | 8,202.37 | 0.84 |
| Median | 35,980.00 | 28.23 | 10.74 | 2,967.62 | 0.08 |
| SD | 52,276.13 | 3.79 | 1.51 | 15,502.44 | 28.04 |
| Min. | -10,800.00 | 18.05 | 3.87 | 9.59 | -11.65 |
| Max. | 1,160,000.00 | 45.12 | 17.06 | 146,866.10 | 4,324.89 |
| Obs. | 60,630 | 60,630 | 60,630 | 60,630 | 59,267 |
| Asian | | | | | |
| Mean | 96,929.72 | 26.19 | 9.90 | 5,296.31 | 0.50 |
| Median | 73,000.00 | 25.47 | 10.17 | 1,683.37 | 0.02 |
| SD | 94,748.99 | 4.38 | 1.87 | 11,230.45 | 33.71 |
| Min. | -14,800.00 | 18.05 | 3.87 | 9.59 | -2.87 |
| Max. | 1,489,000.00 | 45.12 | 17.06 | 153,469.70 | 6,352.28 |
| Obs. | 38,463.00 | 38,463.00 | 38,463.00 | 38,463.00 | 37,773.00 |
| Hispanic | | | | | |
| Mean | 77,100.71 | 28.48 | 10.20 | 9,081.18 | 0.78 |
| Median | 55,000.00 | 28.30 | 10.36 | 2,794.43 | 0.05 |
| SD | 81,808.61 | 4.33 | 1.90 | 17,955.75 | 48.10 |
| Min. | -14,800.00 | 18.05 | 3.87 | 9.59 | -803.54 |
| Max. | 1,760,400.00 | 45.12 | 17.06 | 160,565.40 | 23,813.74 |
| Obs. | 480,664.00 | 480,664.00 | 480,664.00 | 480,664.00 | 474,797.00 |

All statistics computed for counties with both pollution data and income data.

**Table D: Gini Coefficients by Racial Category.**

| Asian Households | | | | | | | |
| --- | --- | --- | --- | --- | --- | --- | --- |
| Year | Obs. | Market  Income | Externality | Market Income  - Externality | Share  PM_2.5_ | Share  O_3_ | Ratio  (Adjusted/  Market) |
| 2011 | 38,463 | 0.461  (0.002) | 0.696  (0.002) | 0.546  (0.002) | 0.803 | 0.197 | 1.183 |
| 2012 | 42,370 | 0.461  (0.002) | 0.697  (0.002) | 0.536  (0.002) | 0.787 | 0.213 | 1.163 |
| 2013 | 44,964 | 0.464  (0.002) | 0.704  (0.002) | 0.538  (0.002) | 0.795 | 0.205 | 1.161 |
| 2014 | 47,720 | 0.463  (0.002) | 0.698  (0.002) | 0.534  (0.002) | 0.783 | 0.217 | 1.152 |
| Hispanic Households | | | | | | | |
| Year | N | Income | Damage | Market Income  - Externality | Share  PM_2.5_ | Share  O_3_ | Ratio  (Adjusted/  Market) |
| 2011 | 55,439 | 0.445  (0.001) | 0.693  (0.002) | 0.577  (0.003) | 0.793 | 0.207 | 1.297 |
| 2012 | 60,078 | 0.442  (0.001) | 0.694  (0.002) | 0.562  (0.002) | 0.782 | 0.218 | 1.271 |
| 2013 | 60,558 | 0.444  (0.002) | 0.693  (0.002) | 0.561  (0.003) | 0.787 | 0.213 | 1.264 |
| 2014 | 64,036 | 0.441  (0.002) | 0.690  (0.002) | 0.551  (0.002) | 0.784 | 0.216 | 1.248 |

**Table E: Sensitivity Analysis.**

| Modeling  Scenario | Obs. | Market  Income | Externality | Market Income  - Externality |
| --- | --- | --- | --- | --- |
| Default | 455,988 | 0.482  (0.001)^A^ | 0.670  (0.000) | 0.682  (0.001) |
| Low VSL |  | 0.482  (0.001) | 0.670  (0.001) | 0.536  (0.001) |
| Alternative  PM Dose-response |  | 0.482  (0.001) | 0.672  (0.001) | 1.032  (0.003) |
| VSL-Income |  | 0.482  (0.001) | 0.669  (0.001) | 0.618  (0.001) |
| Income  Quantile | Default | Low VSL | Alternative  PM Dose-  Response | VSL Income |
| < 20 | -0.074  (0.0005778) | 0.006  (0.0001521) | -0.281  (0.0020039) | -0.036  (0.0003953) |
| 20 – 40 | 0.061  (0.0001949) | 0.073  (0.000165) | 0.0458  (0.0002711) | 0.064  (0.0001782) |
| 40 – 60 | 0.143  (0.0002525) | 0.141  (0.0002108) | 0.158  (0.0003819) | 0.140  (0.0002358) |
| 60 – 80 | 0.255  (0.0003619) | 0.236  (0.0002741) | 0.306  (0.0006889) | 0.245  (0.0003238) |
| 80 – 100 | 0.614  (0.0007293) | 0.545  (0.0005654) | 0.771  (0.0015362) | 0.586  (0.0006504) |

**A = standard errors in parenthesis.**

**Table F: Average PM_2.5_ Levels by Income Quantile.**

| Income Quantiles | PM_2.5_  (se) | Difference from:  Under 20^th^  (se) | Difference  from:  40^th^ - 20^th^  (se) | Difference from:  60^th^ - 40^th^  (se) | Difference from:  80^th^ - 60^th^  (se) |
| --- | --- | --- | --- | --- | --- |
| 20^th^ | 10.40^A^  (0.004) |  |  |  |  |
| 40^th^ – 20^th^ | 10.33  (0.005) | 0.078***  (0.006) |  |  |  |
| 60^th^ – 40^th^ | 10.24  (0.004) | 0.164***  (0.006) | 0.084***  (0.006) |  |  |
| 80^th^ – 60^th^ | 10.15  (0.004) | 0.254***  (0.006) | 0.175***  (0.006) | 0.091***  (0.006) |  |
| > 80^th^ | 10.08  (0.004) | 0.324***  (0.006) | 0.241***  (0.006) | 0.157***  (0.006) | 0.066***  (0.006) |

**A = all values in table A.6 expressed in ug/m^3^**
