# Supplementary figures and images for: The distribution of income is worse than you think: Including pollution impacts into measures of income inequality
Source: PLoS One. 2018 Mar 21;13(3):e0192461. doi: 10.1371/journal.pone.0192461 (PMC5862398; doi:10.1371/journal.pone.0192461)

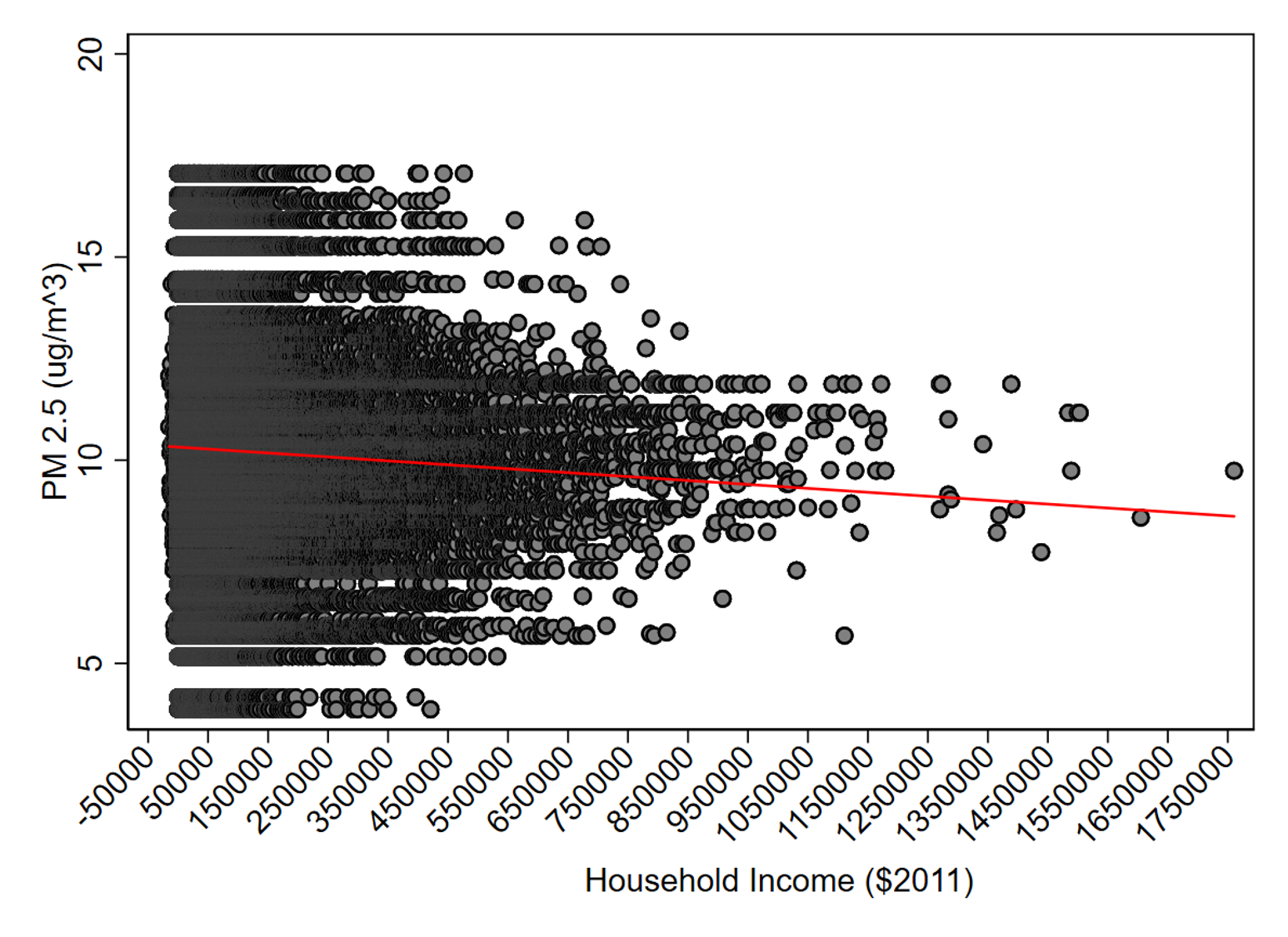

Supplement: S1 Fig — (TIF) [file pone.0192461.s002.tif]

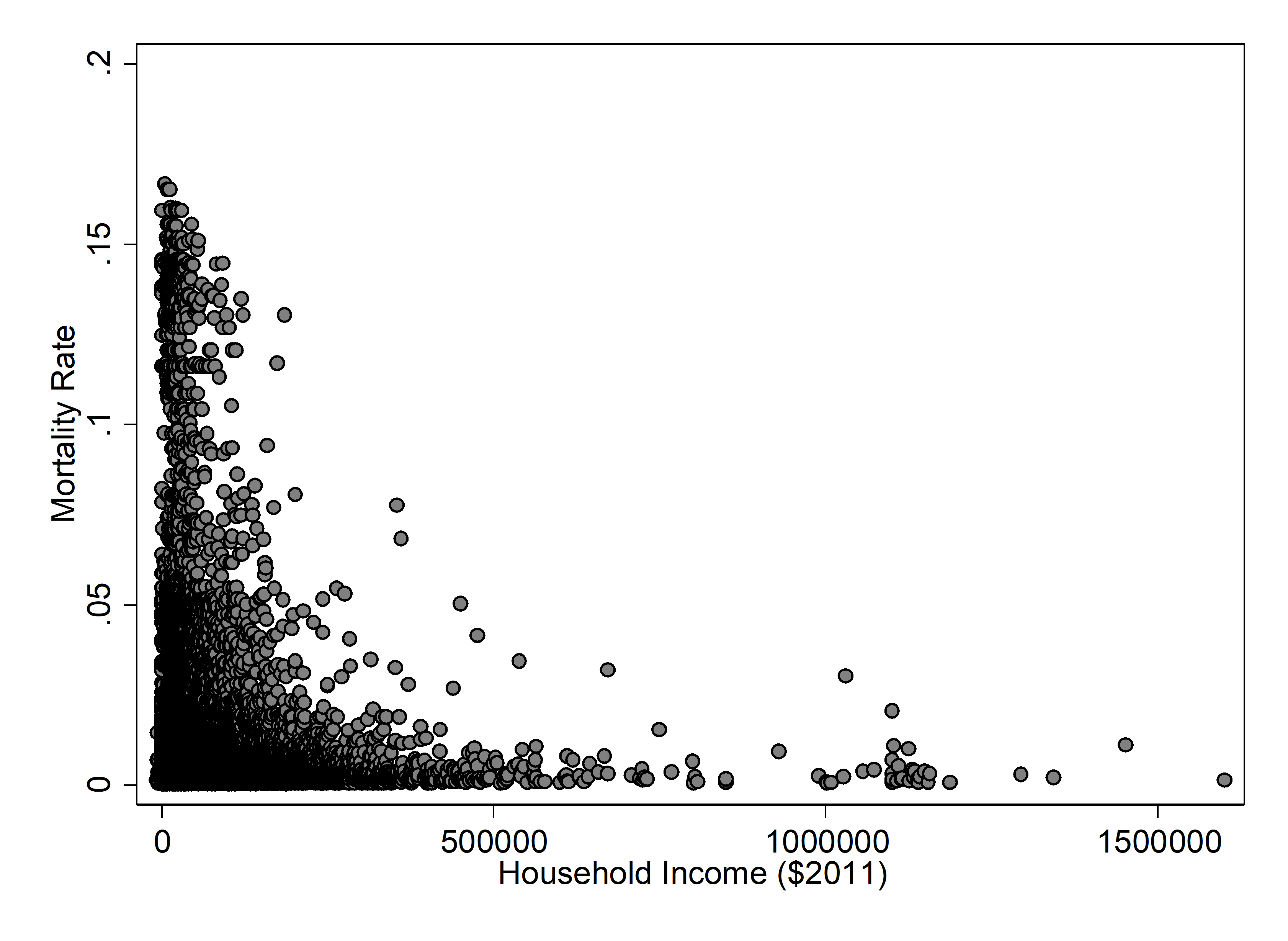

Supplement: S2 Fig — (TIF) [file pone.0192461.s003.tif]

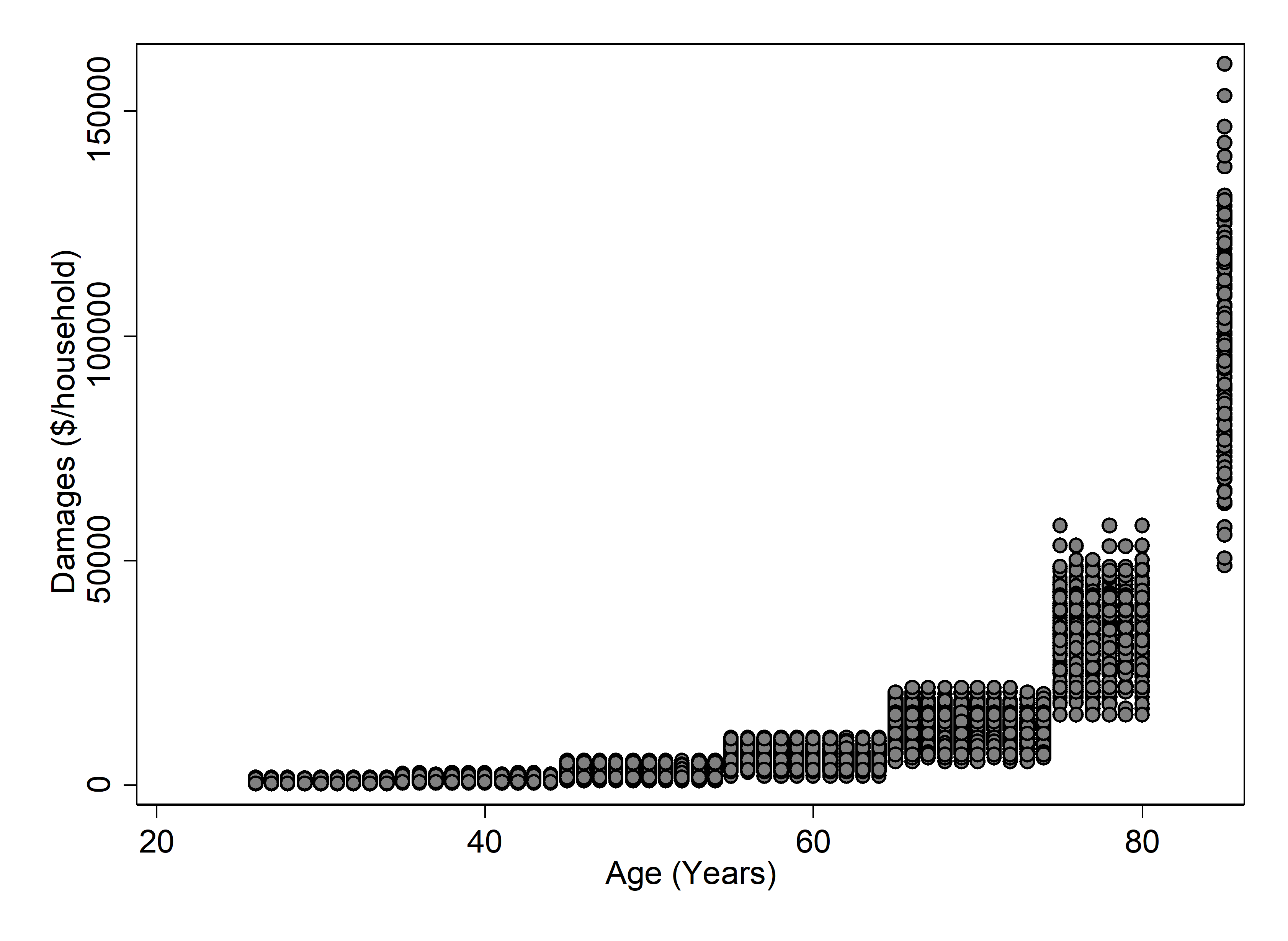

Supplement: S3 Fig — (TIF) [file pone.0192461.s004.tif]

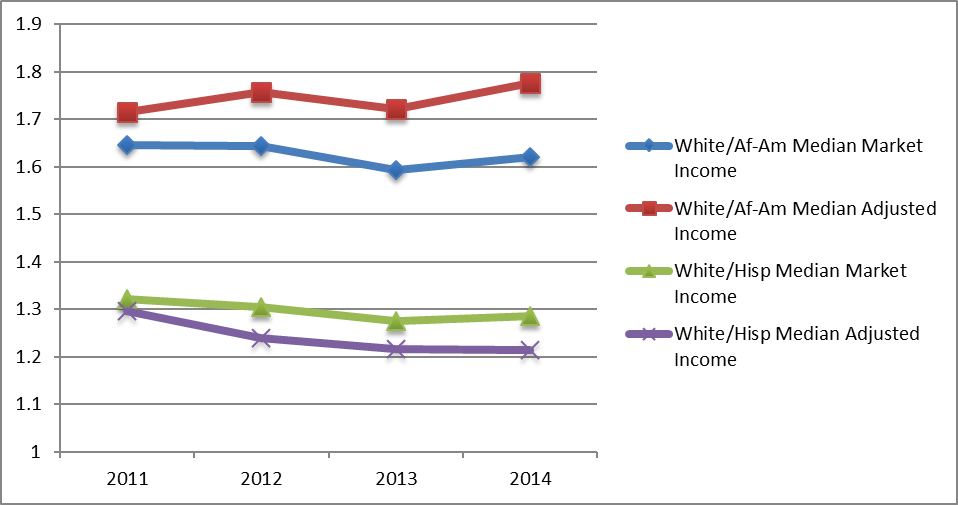

Supplement: S4 Fig — (TIF) [file pone.0192461.s005.tif]
